# Analysis of gasoline-related pollutant exposures and risks in California between 1996 and 2014

**DOI:** 10.1038/s41370-023-00615-0

**Published:** 2023-12-08

**Authors:** Daniel Sultana, Sara Hoover

**Affiliations:** Office of Environmental Health Hazard Assessment, California Environmental Protection Agency, Oakland, CA USA

**Keywords:** Gasoline-related air pollutants, Emission trends, Exposure assessment, Screening health risk assessment, MTBE

## Abstract

**Background:**

Gasoline-powered vehicles and equipment are an important source of air pollution in California. Many gasoline-related pollutants pose significant health concerns. The California Air Resources Board strictly regulates the state’s gasoline formulation and vehicle emissions.

**Objective:**

To investigate exposure trends for gasoline-related air pollutants between 1996 and 2014, capturing the period before and after the removal of methyl t-butyl ether (MTBE).

**Methods:**

We identified gasoline-related chemicals with known or suspected health concerns and adequate ambient air monitoring data. Average exposures to the general public were estimated from 1996 to 2014 in five major air basins and statewide. We determined the fractions of exposures attributable to gasoline use and evaluated cancer and non-cancer risks for chemicals with available cancer potencies and health reference values.

**Results:**

We found that average gasoline-attributable cancer risks for the general California population from the most highly emitted carcinogens (acetaldehyde, benzene, 1,3-butadiene, and formaldehyde) declined by over 80% between 1996 and 2014. This decline occurred despite roughly constant statewide gasoline sales, an increase in vehicle miles traveled, and an approximately 10% increase in vehicle registrations over this same period. Naphthalene, measured as a volatile organic compound (VOC), was the most abundant gasoline-related polycyclic aromatic hydrocarbon (PAH). From 1996 to 2014, gasoline-attributable cancer risks for naphthalene were estimated to drop approximately threefold in the South Coast Air Basin. Exposures to gasoline-related chemicals associated with non-cancer health effects, such as chronic respiratory toxicity or neurotoxicity, were generally below levels of concern. The exception was acrolein, with gasoline-related exposures in 2014 estimated to be high enough to pose risks for respiratory toxicity.

**Impact statement:**

Our historical analysis demonstrated the success of California’s regulatory efforts to reduce gasoline-related air pollutant exposures and risks to the general public. New efforts are focused on addressing gasoline-related and other air pollution in heavily impacted communities affected by multiple environmental and social stressors.

## Introduction

Emissions from cars have declined over the past 30 years due to substantial improvements in vehicle emission controls and the introduction of cleaner burning gasoline [1]. In spite of this progress, traffic-related air pollution remains a public health concern [2]. A large number of studies have linked exposure to traffic and traffic-related air pollution in California to a range of negative health outcomes, such as asthma [3–9]; decreased lung function in asthmatic children and adults [10, 11]; neurodevelopmental disorders [12]; preterm births [13, 14]; low birth weight [15, 16]; pregnancy loss and infant mortality [17–19]; heart disease [20–22]; and overall mortality [23–25]. The population of California has grown and the number of on-road vehicles has increased as well [26–28]. Traffic-related public health impacts will remain an important factor in setting state air pollution policies.

California has a long history of taking action to improve air quality. State regulations to address vehicle emissions have included setting limits on tailpipe emissions, requiring new vehicle technology, and controlling fuel composition and other fuel properties. California introduced reformulated gasoline in 1992 (Phase 1) and 1996 (Phase 2), with the goal of reducing evaporative emissions, cutting emissions of criteria air pollutants and toxic air contaminants (e.g., benzene) and lowering the ozone forming potential of gasoline vehicle emissions [29]. Both Phase 2 and federal reformulated gasoline regulations included requirements designed to improve the combustion efficiency of gasoline and reduce smog-forming and toxic emissions [30, 31]. As part of meeting these requirements refiners added an oxygenate, favoring methyl t-butyl ether (MTBE) as the lowest cost option.

Governor Gray Davis issued an Executive Order in 1999 to remove MTBE from gasoline, which was prompted by potential impacts to groundwater and surface water from leaking underground storage tanks. The Order also directed the California Air Resources Board (CARB), the State Water Resources Control Board (SWRCB), and the Office of Environmental Health Hazard Assessment (OEHHA) to evaluate the potential impacts of replacing MTBE with ethanol [32]. This included modeling selected contaminant levels in ambient air in the South Coast Air Basin based on ethanol’s proposed use in gasoline and an assessment of associated potential health risks. The analysis predicted no substantial differences in the health impacts of harmful air pollutants between the different fuel types evaluated. MTBE was banned from California gasoline at the end of 2003 and Phase 3 gasoline was introduced. The Phase 3 regulations were designed to maintain the air quality benefits of Phase 2 gasoline without the use of MTBE, and allowed ethanol to be added as an oxygenate [33].

As a proactive measure to reduce the chances of another MTBE scenario, OEHHA and other departments in the California Environmental Protection Agency (CalEPA) were funded by the state to track potential health and environmental impacts associated with changing gasoline formulations over time. As part of this effort, OEHHA undertook a comprehensive exposure assessment and health risk analysis of gasoline use in California.

We first investigated whether it would be possible to assess gasoline-related mixtures instead of evaluating individual gasoline components one by one. This included convening workshops to explore approaches for evaluating human health effects from exposure to gasoline-related mixtures [34]. We also reviewed available toxicology literature on gasoline exhaust emissions (see for example [35–37]) and publications on complex mixture assessment (see for example [38–44]). The results of our literature research and consultation with experts indicated that existing data were insufficient for evaluating the particular gasoline formulation used in California as a mixture. Further, assessing exposures and risks for complex mixtures is an ongoing area of research and continues to pose unresolved scientific challenges. These include inadequate toxicological data for real-world mixtures, or even for a large proportion of individual chemicals contained in these, and an incomplete picture of the aggregate multi-source and multi-pathway exposures to mixtures. We determined that the most practical approach for evaluating risks associated with gasoline use was to assess individual gasoline-related chemicals of concern. Health Canada came to a similar conclusion and adopted a chemical-by-chemical approach for their health assessment of gasoline exhaust [45].

We carried out a comprehensive hazard evaluation of gasoline-related pollutants and estimated exposures and health risks for selected chemicals based on data from 1996 to 2014 [46]. The purpose of this historical analysis was to assess gasoline-related air pollutant exposures and associated health risks before and after the removal of MTBE and establish a baseline for evaluating future changes in fuel formulations. This included reviewing emissions from gasoline-related sources to identify gasoline-related VOCs and PAHs and related compounds (nitro-PAHs, oxo-PAHs and selected other polycyclic matter). Available monitoring data were used to generate population-weighted average ambient air concentrations in a particular California air basin (e.g., South Coast) and statewide for the gasoline-related pollutants. A unique aspect of our analysis was determining the gasoline-attributable fractions of the ambient air concentrations. A screening level cancer and non-cancer risk assessment was carried out for the gasoline-related chemicals with health reference values. This involved using the average population-weighted gasoline-attributable concentrations as exposure estimates and applying default assumptions to calculate risks. Here we describe the main findings of our hazard evaluation, exposure estimation, and risk assessment and identify areas where more research is needed.

## Materials and methods

### Emission inventory

CARB provided data extracts from the Emission Inventory (1996–2012) that included annual estimates of primary emissions from each source aggregated by air basin. Primary emissions are those directly emitted from a source and do not account for chemicals formed through secondary atmospheric reactions. CARB assigns a “material code” to each type of source. Gasoline-related sources were identified using material code 1100. We used the Emission Inventory data to track trends in gasoline-related emissions, identify specific chemicals associated with gasoline-related sources, and calculate the fractions of those chemicals that came from gasoline-related sources. This time period was selected to cover the years before and after the phase-out of MTBE at the end of 2003. At the time of our study, the latest emission estimates available were for 2012. The Emission Inventory catalogs a range of air pollution sources, including mobile sources (e.g., cars), stationary sources (e.g., power plants), area-wide sources (e.g., fireplaces and farming activities), and natural sources (e.g., plants, trees, and wildfires). For each source, the Emission Inventory contains estimated primary emissions of total organic gases (TOG), carbon monoxide, NO_x_, sulfur oxides, and particulate matter.

Total organic gases (TOG) are defined as VOCs and lower volatility organic compounds [47]. The Emission Inventory links each source to a “speciation profile,” which provides the chemical composition of the TOG from that source. We used the speciation profiles and the gasoline-related material code (1100) to determine emission estimates for individual VOCs from gasoline and non-gasoline sources.

### Hazard identification for gasoline-related chemicals

We used the Emission Inventory to rank VOCs emitted from gasoline-related sources by annual statewide primary emissions. This ranking was used as an indication of exposure potential to help prioritize the gasoline-related VOCs for exposure and health risk assessment.

We also evaluated the gasoline-related VOCs for potential toxicity, with a focus on chronic effects that are amenable to screening-level risk assessment based on annual average exposures. Health endpoints examined included carcinogenicity, chronic respiratory toxicity, reproductive/developmental toxicity and neurotoxicity. We did not address potential health effects from short-term peak exposures, or other types of chemical hazards like explosivity and flammability.

Known carcinogens were identified primarily from the Proposition 65 list of chemicals known to the state to cause cancer [48]. Acrolein was identified by the International Agency for Research on Cancer as a Group 2 A carcinogen and crotonaldehyde as a Group 2B carcinogen [49]. OEHHA developed a Public Health Goal for MTBE based on its carcinogenic effects [50] and provided the cancer potency to CARB to support its evaluation of MTBE as a Toxic Air Contaminant.

Benzaldehyde was included as a suspected carcinogen in Table 1 based on the National Toxicology Program (NTP) [51] finding of some evidence of carcinogenic activity in male and female mice.

**Table 1:** Gasoline-related VOCs: Exposure indicator and health hazard screening results.

Gasoline-related VOC	2012 Primary emissions rank^a^	Selected toxicities^b^				Screening risk assessment conducted	
		Carcinogen	Developmental or reproductive toxicant	Respiratory toxicant	Neurotoxicant	Cancer risk	Non-cancer Hazard Quotient
*Top 25 most highly emitted*							
Isopentane	1						
Ethanol^c^	2						
Toluene	3		✓		✓		✓
Methane	4						
Ethylene	5						
2-Methylpentane	6						
*n*-Pentane	7						
2,2,4-Trimethylpentane	8						
Xylenes	9, 22, 54^d^			✓	✓		✓
*n-*Butane	10						
3-Methylpentane	11						
Methylcyclopentane	12						
Propylene	13			✓			✓
2,3-Dimethylpentane	14						
Benzene	15	✓	✓			✓	✓
3-Methylhexane	16						
Acetylene	17						
*n-*Hexane	18		✓		✓		✓
2,3-Dimethylbutane	19						
2-Methylhexane	20						
Formaldehyde	21	✓		✓		✓	✓
Ethylbenzene	23	✓	✓			✓	✓
2,4-Dimethylpentane	24						
Isobutene	25			S^e^			✓
*Lower-emitted VOCs with toxicity concerns*							
Trimethylbenzenes	27, 46, 71^f^				✓		✓
1,3-Butadiene	45	✓	✓			✓	✓
Acetaldehyde	47	✓		✓		✓	✓
Benzaldehyde	62	S		S			
*m-*Tolualdehyde	63			S			
Methanol	75		✓				
Isoprene	91	✓				✓	
Styrene	93	✓			✓	✓	✓
Acrolein	94	✓^g^		✓			✓
Naphthalene	95	✓		✓		✓	✓
Propionaldehyde	124			S			
2-Butenal (crotonaldehyde)	128	✓^g^		S			
Cumene (isopropylbenzene)	140	✓					
3-Methylbutanal (isovaleraldehyde)	149			S			
*n*-Butanal (butyraldehyde)	161			S			
Hexaldehyde	177			S			
Methyl *t*-butyl ether (MTBE)	362	✓^h^				✓	

Table Notes:^a^ 2012 Emissions Rank based on statewide primary emissions from gasoline-related sources.

^b^Based on Proposition 65 list or target organs of inhalation reference values from OEHHA or US EPA unless otherwise noted.

^c^Under Proposition 65, alcoholic beverages are listed as a carcinogen and ethanol in alcoholic beverages is listed as a developmental toxicant. Ethanol in alcoholic beverages is also identified as a Group 1 carcinogen by IARC. No chronic non-cancer inhalation reference values (i.e., cREL or RfC) are available for ethanol.

^d^Xylene emission ranks: 9 (m-xylene), 22 (o-xylene), 54 (p-xylene).

^e^Suspected based on published studies or structural analogy.

^f^Trimethylbenzene emission ranks: 27 (1,2,4-TMB), 46 (1,3,5-TMB), 71 (1,2,3-TMB).

^g^Identified as carcinogen by IARC [49].

^h^OEHHA developed a Public Health Goal for MTBE based on its carcinogenic effects [50].

Known non-cancer health effects were based on the target organs identified by OEHHA in developing chronic Reference Exposure Levels (cRELs) [52] and by the US Environmental Protection Agency (US EPA) in developing reference concentrations (RfCs) [53, 54]. We also used the Proposition 65 list to identify known reproductive toxicants, with endpoints including male and female reproductive toxicity and developmental toxicity [48].

Isobutene was identified as a suspected respiratory toxicant based on findings of degeneration of the olfactory epithelium in male and female rats and mice and hyaline degeneration of the respiratory epithelium in male and female mice in the NTP inhalation bioassays [55]. Benzaldehyde was identified as a suspected respiratory toxicant based a short-term inhalation study that found goblet cell metaplasia in the respiratory epithelium lining the nasal septum in male rats [56]. The other lower-emitted aldehydes in Table 1 were flagged as suspected respiratory toxicants based on structural analogy to known toxic members of that chemical class.

### Methods for calculating gasoline-attributable fractions

For all VOCs identified from the Emission Inventory, we estimated the fractions of primary emissions that came from gasoline-related sources (identified by material code 1100) statewide. Primary emissions include both tailpipe and evaporative emissions. Using data from 1996 and 2012, statewide gasoline-attributable fractions were calculated for each VOC as follows:

$${{{{{\rm{Gasoline}}}}}}-{{{{{{\rm{attributable}}}}}}\; {{{{{\rm{fraction}}}}}}}=\frac{{{{{\rm{Emissions}}}}}_{{{{\rm{gas}}}}}}{{{{{\rm{Emissions}}}}}_{{{{\rm{all}}}}}}$$

where Emissions_gas_ and Emissions_all_ were the tons of primary VOC emissions statewide from gasoline-related sources and from all sources, respectively. For selected VOCs, gasoline-attributable fractions were also calculated based on the emissions reported for the five most populated air basins (South Coast [SC], San Diego [SD], San Francisco Bay Area [SF], San Joaquin Valley [SJV] and Sacramento Valley [SV]) and statewide for all years from 1996 to 2012.

For some gasoline-related VOCs, such as acetaldehyde, acrolein and formaldehyde, substantial portions of the ambient air concentrations are attributable to secondary atmospheric formation. Under contract with OEHHA, Carter modeled the secondary atmospheric reactions and estimated factors called “direct formation potentials” [57]. We used these in conjunction with the Emission Inventory to calculate the tons of the chemicals formed through secondary reactions in the atmosphere. We carried out this calculation for the chemicals or chemical groups with available direct formation potentials, which included:

- Acetaldehyde, formaldehyde, aromatic aldehydes (e.g., benzaldehyde), and higher aldehydes (e.g., propionaldehyde)
- Acrolein
- Cresols
- Peroxyacetyl nitrate (PAN), higher saturated acyl peroxynitrates, aromatic peroxynitrates (e.g., peroxybenzoyl nitrate), and unsaturated PAN analogs (e.g., the PAN analog formed from methacrolein).

For chemicals with substantial secondary formation, the gasoline-attributable fraction was calculated as:

$${{{{{\rm{Gasoline}}}}}}-{{{{{{\rm{attributable}}}}}}\, {{{{{\rm{fraction}}}}}}}=\frac{{{{{\rm{Emissions}}}}}_{{{{{{\rm{gas}}}}}}}+{{{{\rm{Secondary}}}}\,{{{\rm{formation}}}}}_{{{{\rm{gas}}}}}}{{{{{\rm{Emissions}}}}}_{{{{\rm{all}}}}}+{{{{\rm{Secondary}}}}\,{{{\rm{formation}}}}}_{{{{{\rm{all}}}}}}}$$

where Emissions_gas_ and Emissions_all_ are as defined above; Secondary formation_gas_ and Secondary formation_all_ are the tons of the VOC formed through secondary reactions of gasoline-related emissions and emissions from all sources, respectively.

### Ambient air monitoring data

Data from California’s monitoring network for TACs were used to estimate ambient exposures in the South Coast Air Basin and statewide for acetaldehyde, acrolein, benzene, 1,3-butadiene, formaldehyde, MTBE, toluene and xylene isomers. Non-methane organic compound (NMOC) data from the Photochemical Assessment Monitoring Stations (PAMS) network were used to estimate exposures in the South Coast Air Basin for cumene, ethylbenzene, *n*-hexane, propylene, styrene and trimethylbenzene isomers. Isobutene concentrations in the South Coast Air Basin were modeled from more limited PAMS data collected primarily during the summer of 2001 and 2002. Benzaldehyde concentrations were modeled based on data collected by the Desert Research Institute (DRI) at three monitoring sites in the South Coast Air Basin (Azusa, Burbank and North Main) in the summer of 1996 [58]. Naphthalene concentrations in the South Coast Air Basin were modeled on data collected from National Air Toxic Trends Stations (NATTS) from 2007 to 2014. For ambient air data on particle-bound PAHs we accessed multiple sources, including California’s monitoring network for TACs, NATTS, Multiple Air Toxics Study III (MATES III) report, and other scientific publications.

### Methods for calculating population-weighted annual average ambient air concentrations for selected VOCs

Our approach for estimating the population-weighted average concentration of a VOC in a specific air basin was based on the earlier assessment of ethanol as a replacement oxygenate that was carried out by CARB and OEHHA [32]. The approach included the following steps:

- Estimating the annual average concentration of a chemical at as many air monitoring sites as possible within the air basin, using modeling to supplement the measured values.
- Estimating a weighted annual average at each census tract based on the monitoring site estimates from the previous step. The weights were proportional to the inverse square distance from the census tract to the monitoring sites. Only sites within 30 km of a census tract centroid were included in the average.
- Calculating a population-weighted average of the census tract estimates in a particular air basin. The weights were proportional to the census tract populations.

Naphthalene, isobutene and benzaldehyde had limited ambient data and required more extensive modeling to calculate population-weighted annual averages in the South Coast Air Basin (see section on ambient air monitoring data above and [46] for details).

The population-weighted average ambient air concentration of a chemical was multiplied by the gasoline-attributable fraction to obtain the gasoline-attributable population-weighted average ambient air concentration (hereafter referred to as the “gasoline-attributable concentration”). For the complete set of gasoline-attributable concentrations, refer to the Chemical Profiles in [46].

### Methods for estimating cancer risks for gasoline-related VOCs

Cancer risks associated with general population exposures to gasoline-related VOCs (including the volatile PAH naphthalene) were estimated for the South Coast Air Basin and statewide in accordance with OEHHA guidance for assessing airborne emissions [59]. The calculation took into account early-in-life sensitivity, which incorporates age sensitivity factors and 95th percentiles of age-specific breathing rates [59]. We used cancer potencies derived by OEHHA [59, 60]. The equations used for estimating cancer risk are shown below.

#### Average daily dose (ADD) by age range

$${{{{{{\rm{ADD}}}}}}}_{{{{{{{\rm{age}}}}}}}\,{{{{{{\rm{range}}}}}}}}={{{{{\rm{GC}}}}}}\times{{{{{{\rm{BR}}}}}}}_{{{{{{{\rm{age}}}}}}}\,{{{{{{\rm{range}}}}}}}}$$

where GC is the gasoline-attributable concentration and BR_age range_ is the 95th percentile of the body-weight adjusted breathing rate for the age range.

#### Gasoline-attributable cancer risk

$${{{{{\rm{Cancer}}}}}}\,{{{{{\rm{risk}}}}}}=\mathop{\sum}\limits_{{{{{{\rm{age}}}}}}\,{{{{{\rm{range}}}}}}}{{{{{{\rm{ADD}}}}}}}_{{{{{{\rm{age}}}}}}\,{{{{{\rm{range}}}}}}}\,\times\,{{{{{\rm{CPF}}}}}}\,\times\,{{{{{{\rm{ASF}}}}}}}_{{{{{{\rm{age}}}}}}\,{{{{{\rm{range}}}}}}}\,\times\,{{{{{\rm{Fraction}}}}}}\,{{{{{\rm{of}}}}}}\,{{{{{\rm{lifetime}}}}}}$$

where ADD_age range_ is the average daily dose for the age range, CPF is the cancer potency factor, ASF_age range_ is the age sensitivity factor for the age range, and fraction of lifetime is the length of the age range divided by 70. For an example cancer risk calculation and the CPFs, refer to the Supplementary Material (SM).

### Methods for estimating non-cancer hazard quotients

Non-cancer hazard quotients were calculated as the ratio of a chemical’s gasoline-attributable concentration (GC) to its non-cancer health reference value, which in most cases was the OEHHA chronic Reference Exposure Level (cREL).

$${{{{{\rm{Gasoline}}}}}}-{{{{{{\rm{attributable}}}}}}\; {{{{{\rm{hazard}}}}}}\; {{{{{\rm{quotient}}}}}}}=\frac{{{{{{\rm{GC}}}}}}}{{{{{{\rm{cREL}}}}}}}$$

The cRELs are air concentrations of chemicals at or below which no adverse health effects are anticipated to occur following long-term exposure [59]. Each cREL is associated with hazard index target organs or organ systems, such as the respiratory system and nervous system. Refer to the SM for the non-cancer health reference values used in the assessment. We calculated non-cancer hazard quotients for chemicals with cRELs that had sufficient ambient air monitoring data to estimate population-weighted exposure concentrations. The most comprehensive dataset was available from the South Coast Air Basin. We also calculated statewide non-cancer hazard quotients when possible.

Multiple gasoline-related pollutants affected the respiratory system and nervous system, so we also examined the hazard indices for these two target systems (i.e., the sums of the hazard quotients for the individual chemicals affecting the relevant system). For the respiratory toxicity hazard index, acetaldehyde, acrolein, formaldehyde, naphthalene, propylene, toluene, and xylenes were included. For the nervous system hazard index, n-hexane, styrene, toluene, and xylenes were included.

## Results

### VOC emissions from gasoline sources

**Fig. 1:**
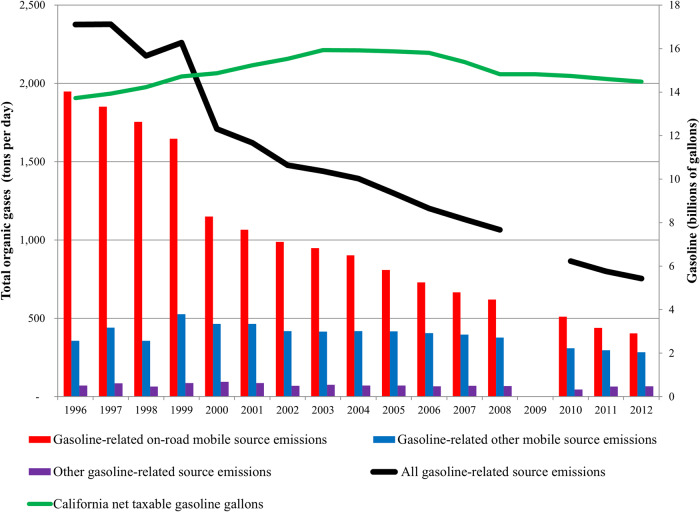
Statewide trends in gasoline-related emissions and gasoline use. Total organic gases (left axis) from all gasoline-related sources and component categories are plotted for the years 1996 to 2012. Net taxable gasoline gallons sold in California (right axis) are also shown for this same period.

Estimated primary emissions of total organic gases from gasoline-related sources declined by 68% statewide between 1996 and 2012 (see Fig. 1). This significant reduction is attributable primarily to the decline in on-road mobile source emissions that occurred even while gasoline sales remained steady and California’s population continued to grow [26, 61]. The 10 chemicals that contributed most to this decline were isopentane, methyl t-butyl ether (MTBE), methane, n-butane, toluene, ethylene, n-pentane, m-xylene, 2-methylpentane, and isobutene. By 2012, emissions from on-road motor vehicles had decreased so much that they were approaching levels emitted by other gasoline-related mobile sources, including lawn and garden equipment, recreational boats and off-road vehicles. Less apparent in Fig. 1 is the decline in emissions from these other mobile sources, dropping almost in half between 1999 and 2012.

We identified more than 350 VOCs in primary emissions from gasoline-related sources based on the 2012 Emission Inventory. Table 1 lists gasoline-related VOCs we identified as being of concern for exposure and/or health hazards. The top 25 most emitted VOCs made up more than 75% of all gasoline-related TOG emissions in 2012. Isopentane has consistently been the most highly emitted chemical from gasoline-related sources. Between 1996 and 2003, the second most highly emitted gasoline-related VOC was MTBE. In 2004, MTBE was phased out and MTBE emissions dropped to nearly zero. Ethanol replaced MTBE as the oxygenate of choice and by 2012 it became the second most highly emitted gasoline-related VOC. After accounting for secondary atmospheric formation, PAN, formaldehyde, and acetaldehyde were in the top five most highly emitted VOCs based on 2012 data.

Among the top 100 VOCs emitted in 1996, 98 had lower gasoline-related emissions in 2012 (refer to the SM for complete details). Reductions in emissions from on-road gasoline-powered vehicles accounted for the bulk of the decline. Two VOCs with significant health concerns, benzene and 1,3-butadiene, had large declines in gasoline-related emissions, 74% and 75%, respectively. Ethanol showed a large increase in gasoline-related emissions between 1996 (2.2 tons/day) and 2012 (39 tons/day), which was expected due to its use as a replacement oxygenate for MTBE.

### Hazard identification – VOCs in primary emissions

Table 1 summarizes the known or suspected toxicities for gasoline-related VOCs. A number of highly emitted hydrocarbons in Table 1 are not of significant concern for human health as the parent compound (e.g., isopentane); however, their atmospheric transformation products (e.g., acetaldehyde) can pose health hazards.

Ethanol has only been identified as a carcinogen or developmental toxicant in association with consumption of alcoholic beverages (see footnote 3 to Table 1 for more details), and no chronic non-cancer inhalation reference values (i.e., cREL or RfC) are available.

The most significant toxicants in the top 25 gasoline-related VOCs include the BTEX compounds (i.e., benzene, rank 15; toluene, rank 3; ethylbenzene, rank 23; and xylenes [(*m*-xylene, rank 9; *o*-xylene, rank 22]), with endpoints including cancer, developmental and reproductive toxicity, respiratory toxicity, and neurotoxicity, depending on the specific chemical. Other highly emitted gasoline-related chemicals of note for toxicity include propylene (rank 13; respiratory toxicant), *n*-hexane (rank 18; male reproductive toxicant and neurotoxicant), and formaldehyde (rank 21; carcinogen and respiratory toxicant).

Table 1 also includes VOCs that had lower levels of primary emissions, but still pose potential concerns due to toxicity. Some of these also have substantial secondary formation. The lower-emitted VOCs of concern included 1,3-butadiene (rank 45), acetaldehyde (rank 47), acrolein (rank 94), benzaldehyde (rank 62), isoprene (rank 91), styrene (rank 93), naphthalene (rank 95), and cumene (rank 140).

Table 1 indicates the VOCs for which we were able to complete a screening level risk assessment. Of the chemicals with known or suspected toxicities, the majority that could not be assessed were the lower ranked aldehydes, which did not have available health reference values and/or sufficient ambient air data for modeling.

### Hazard identification – atmospheric transformation products

Many gasoline-related VOCs undergo atmospheric transformations to form secondary products [62], and these can pose potential toxicity concerns. We had sufficient data on emissions, ambient air concentrations, and health reference values to conduct the screening risk assessment for only a few atmospheric products, including acetaldehyde, acrolein and formaldehyde. Some of the unassessed atmospheric products of concern included additional aldehydes (e.g., benzaldehyde, crotonaldehyde, hexaldehyde, tolualdehyde), dicarbonyls (e.g., diacetyl, glyoxal, ethylglyoxal, methylglyoxal, malonaldehyde), cresols, furan, PAN analogs, PAH products (e.g., 2-formylcinnamaldehyde, naphthols, quinones) and nitro-PAHs.

### Gasoline-attributable fractions for VOCs

Table 2 presents the 2012 regional and statewide gasoline-attributable fractions for VOCs identified as having health concerns. These fractions ranged from less than 1% to over 90% and varied across the five air basins. As an example regional difference, the fractions for 1,3-butadiene were 47% in the South Coast Air Basin and 24% in the San Joaquin Valley Air Basin. Sources of 1,3-butadiene in both regions included mobile sources, plastic product manufacturing, and wildfires, with proportionally more gasoline-related mobile sources in the South Coast.

**Table 2:** Regional and statewide gasoline-attributable fractions based on 2012 emissions for chemicals with health concerns.

Chemical	South Coast Air Basin	San Diego Air Basin	San Francisco Bay Area Air Basin	San Joaquin Valley Air Basin	Sacramento Valley Air Basin	Statewide
*Gasoline-related VOCs based on primary emissions only*						
Benzene	78%	76%	63%	40%	58%	63%
1,3-Butadiene	47%	33%	58%	24%	19%	26%
Cumene	51%	50%	46%	40%	44%	46%
Ethanol	34%	36%	31%	6%	29%	22%
Ethylbenzene	84%	78%	80%	70%	77%	78%
n-Hexane	63%	69%	53%	46%	45%	57%
Isobutene	90%	92%	90%	72%	86%	86%
Isoprene	<1%	<1%	<1%	<1%	<1%	<1%
Methanol	3%	1%	2%	<1%	<1%	1%
Propylene	49%	34%	55%	21%	18%	22%
Styrene	15%	3%	22%	8%	9%	12%
Toluene	51%	40%	52%	40%	46%	48%
1,2,3-Trimethylbenzene	67%	66%	67%	57%	62%	64%
1,2,4-Trimethylbenzene	81%	77%	79%	71%	75%	77%
1,3,5-Trimethylbenzene	90%	88%	89%	78%	85%	86%
m- and p-Xylene	67%	65%	72%	64%	63%	66%
o-Xylene	55%	48%	61%	49%	48%	52%
*Gasoline-related VOCs based on primary emissions and secondary atmospheric formation*						
Acetaldehyde	43%	27%	31%	8%	8%	13%
Acrolein	58%	33%	32%	14%	25%	28%
Benzaldehyde	74%	53%	74%	53%	64%	68%
Formaldehyde	35%	19%	23%	9%	6%	11%
Propionaldehyde, butyraldehyde, and other higher aldehydes	33%	19%	24%	8%	7%	11%
*Atmospheric transformation products of gasoline-related chemicals*						
Cresols	65%	56%	65%	55%	57%	60%
Nitrophenols and aromatic nitro-compounds	60%	44%	60%	40%	51%	53%
Alkyl nitrates	43%	23%	28%	13%	8%	13%
Peroxyacetyl nitrate (PAN)	44%	26%	29%	10%	8%	14%
Higher PAN analogs (higher saturated acyl peroxynitrates)	47%	29%	33%	13%	9%	16%
Unsaturated PAN analogs	9%	2%	3%	1%	1%	1%
Peroxybenzoyl nitrate and other aromatic PAN analogs (aromatic acyl peroxynitrates)	54%	30%	58%	38%	45%	49%
*Gasoline-related PAHs in the Emission Inventory*						
Naphthalene	56%	23%	43%	31%	22%	33%

For the chemicals identified as being gasoline-related, the percentage of total primary emissions from gasoline-related sources was 32% in 1996 and 11% in 2012. The specific time trends in gasoline-attributable fractions were different for each chemical, primarily due to changes in emissions from non-gasoline sources. For example, non-gasoline emissions of benzene (e.g., industrial processes, other mobile sources, and waste disposal) were roughly constant between 1996 and 2012, while non-gasoline emissions of toluene (e.g., cleaning and surface coatings and solvent evaporation) declined. In addition, the approach used by CARB to estimate natural source emissions (e.g., wildfires) was changed in 2002 and later years, which affected the temporal trend in gasoline-attributable fractions for VOCs like acetaldehyde and formaldehyde [46].

### Gasoline-attributable air concentrations

Gasoline-attributable ambient concentrations were calculated for VOCs with identified health hazards and available monitoring data. Table 3 summarizes changes between 1996 and 2014 for gasoline-attributable concentrations in the South Coast Air Basin and statewide (where available). In the South Coast Air Basin, gasoline-attributable ambient air concentrations declined by 70 to 90% between 1996 and 2014 for the VOCs we could assess in detail. Statewide gasoline-attributable concentrations also declined by a similar amount between 1996 and 2014. Some chemicals could only be assessed over a shorter time period and are not shown in Table 3. Statewide gasoline-attributable concentrations for acrolein declined by 28% from 2004 to 2014 (the years with acrolein ambient air monitoring data) but were roughly constant in the South Coast Air Basin during the same time period. Statewide gasoline-attributable concentrations of acetaldehyde and formaldehyde declined by 47% and 59% between 2002 and 2014 (the years with stable natural source emission estimates; see discussion above).

**Table 3:** Percentage decline in gasoline-attributable concentrations between 1996 and 2014.

Chemical	South Coast Air Basin	Statewide
Benzene	80%	79%
1,3-Butadiene	92%	89%
Cumene	80%	
Ethylbenzene	81%	
*n*-Hexane	77%	
Isobutene	72%	
Propylene	84%	
Styrene	94%	
Toluene	83%	80%
1,2,3-Trimethylbenzene	90%	
1,2,4-Trimethylbenzene	92%	
1,3,5-Trimethylbenzene	88%	
*m*- and *p*-Xylene	78%	71%
*o*-Xylene	78%	73%

We were not able to generate gasoline-related concentrations for a number of chemicals of interest. For example, many pollutants with health concerns, such as butyraldehyde, crotonaldehyde, hexaldehyde, isovaleraldehyde, propionaldehyde and tolualdehyde, could not be assessed because ambient air monitoring data were insufficient.

### Health risk assessment for VOCs and PAHs

**Fig. 2:**
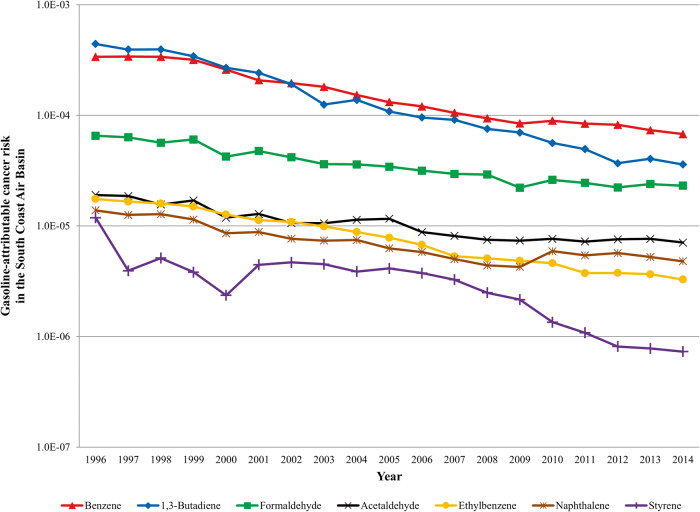
Gasoline-related cancer risks for VOCs in the South Coast Air Basin. Cancer risks based on gasolineattributable population-weighted annual average ambient air concentrations are plotted for benzene, 1,3-butadiene, formaldehyde, acetaldehyde, ethylbenzene, naphthalene, and styrene in the South Coast Air Basin between 1996 and 2014.

Screening level assessments were carried out to estimate average gasoline-attributable cancer risks on a population-weighted basis for selected VOCs. Table 4 compares the gasoline-attributable cancer risks statewide and in the South Coast Air Basin for 1996 and 2014. In the South Coast Air Basin, the combined gasoline-attributable cancer risk from acetaldehyde, benzene, 1,3-butadiene, ethylbenzene, formaldehyde, naphthalene and styrene declined by 84%, from 9.1 × 10^−4^ in 1996 to 1.4 × 10^−4^ in 2014 (Fig. 2). The majority of the gasoline-attributable cancer risk in the South Coast Air Basin came from benzene, 1,3-butadiene and formaldehyde exposures. The naphthalene gasoline-attributable cancer risks, based on modeled ambient concentrations, declined 3-fold between 1996 and 2014.

**Table 4:** Gasoline-attributable cancer risk in the South Coast Air Basin and statewide.

	South Coast Air Basin			Statewide		
	1996	2014	Percent decline	1996	2014	Percent decline
Acetaldehyde^a,b^	1.9E-05	7.1E-06	63%	1.2E-05	2.1E-06	83%
Benzene	3.4E-04	6.8E-05	80%	2.6E-04	5.3E-05	79%
1,3-Butadiene	4.4E-04	3.6E-05	92%	1.8E-04	1.9E-05	89%
Ethylbenzene	1.8E-05	3.3E-06	81%	–	–	–
Formaldehyde^a,b^	6.5E-05	2.3E-05	65%	3.9E-05	6.8E-06	83%
Methyl t-butyl ether (MTBE)	2.1E-05	–	–	1.6E-05	–	–
Naphthalene^c^	1.4E-05	4.8E-06	65%	–	–	–
Styrene	1.2E-05	7.3E-07	94%	–	–	–
Total^d^	9.1E-04	1.4E-04	84%	4.9E-04	8.2E-05	83%

^a^Annual averages concentrations of acetaldehyde and formaldehyde had substantial year to year variability.

^b^Gasoline-attributable fractions for acetaldehyde and formaldehyde were affected by a methodology change in estimating natural source emissions.

^c^Naphthalene ambient air concentrations were modeled. The model was based on naphthalene data collected from 2007 to 2014.

^d^Totals do not include MTBE cancer risk.

Fewer chemicals had sufficient statewide ambient air monitoring data to estimate cancer risks. The combined statewide gasoline-attributable cancer risk for acetaldehyde, benzene, 1,3-butadiene and formaldehyde was 4.9 × 10^−4^ in 1996, declining more than 80% to 8.2 × 10^−5^ by 2014. Statewide, gasoline-attributable cancer risk in 2014 was primarily attributable to benzene and 1,3-butadiene (5.3 × 10^−5^ and 1.9 × 10^−5^, respectively). For comparison, CARB estimated statewide cancer risks from all emission sources of these carcinogens, reporting somewhat lower declines between 1996 and 2007 for benzene (59%) and 1,3-butadiene (66%) [63].

We identified seven gasoline-related particle-bound PAHs that had ambient air data and cancer potencies or potency equivalency factors: benz[a]anthracene, benzo[a]pyrene, benzo[b]fluoranthene, benzo[k]fluoranthene, chrysene, dibenz[ah]anthracene, indeno[1,2,3-cd]pyrene. We were not able to estimate gasoline-attributable fractions for these, so we estimated total cancer risks at two sites in the South Coast Air Basin based on 2014 data. Even without adjusting for gasoline-only exposures, the total cancer risk for these seven PAHs was still about an order of magnitude lower than the estimated gasoline-attributable cancer risk for the volatile PAH naphthalene.

To assess non-cancer risks, we calculated hazard quotients for VOCs with available chronic health reference values and for which we could estimate gasoline-attributable annual average ambient air concentrations in the South Coast Air Basin and statewide. These included acetaldehyde, acrolein, benzene, formaldehyde, n-hexane, naphthalene, propylene, styrene, toluene, trimethylbenzenes, and xylenes. Only acrolein had a clearly elevated hazard quotient, which was for chronic respiratory toxicity. During 2004–2014 (the time period with adequate data for acrolein), the hazard quotient in the South Coast Air Basin varied between 3 and 4, but did not follow a time trend and remained elevated at 3 in 2014. The gasoline-attributable hazard quotient for acrolein based on average statewide exposures was slightly elevated (1.2) in 2014. Acrolein also accounted for the majority of the 2014 respiratory toxicity hazard indices in the South Coast Air Basin and statewide, with a small contribution (<5%) from formaldehyde.

## Discussion

Previous studies have shown that California’s Reformulated Gasoline Program and strict vehicle emission standards, along with fleet turnover have reduced vehicular emissions of many air pollutants [64–68]. Other studies have shown significant downward trends in ambient air concentrations of these air pollutants [58, 69–71]. Our analysis has further documented the success of California’s efforts by showing specific reductions in gasoline-attributable air pollutant exposures and associated health risks. The results of our analysis also supported the findings of the 1999 ethanol evaluation [32] that no substantial changes in harmful air contaminants were expected from its use as a replacement for MTBE.

Despite substantial improvements in air quality, the gasoline-related VOCs benzene and 1,3-butadiene continued to be public health concerns. In 2014, gasoline-attributable cancer risks for benzene and 1,3-butadiene each exceeded 1 × 10^−5^ in the South Coast Air Basin and statewide. As a check on more recent trends, we examined ambient air concentrations between 2014 and 2019 for benzene and 1,3-butadiene, finding declines of about 35% and 40%, respectively. Assuming the other factors in the calculation (e.g., gasoline-attributable fractions) remained similar, the cancer risks associated with exposures to benzene and 1,3-butadiene from gasoline-related sources still would have been greater than 1 × 10^−5^ in 2019.

The respiratory toxicity associated with gasoline-related acrolein exposures also continued to pose a concern. Acrolein is found in primary gasoline-related emissions and is formed via the atmospheric transformation of 1,3-butadiene as its main precursor [62]. It was recently classified by IARC as probably carcinogenic to humans (Group 2A) [49, 72]. The hazard quotients (respiratory system) for acrolein remained elevated in 2014 in the South Coast Air Basin (3) and statewide (1.2). Between 2014 and 2019, ambient air concentrations of acrolein declined by about 35%, but this would not be sufficient to lower the South Coast Air Basin hazard quotient to below 1. Future assessments should expand the cancer risk assessment to address acrolein when a potency is available.

The COVID-19 pandemic substantially altered Californians’ driving habits, so it would be important to reexamine the risks of these gasoline-related VOCs in the coming years. A better understanding of the universe of gasoline-related VOCs would improve any future assessments. Non-targeted analyses of ambient air could be carried out to help identify new chemicals of concern, such as previously unmeasured atmospheric transformation products. Pilot field studies of selected chemicals could be used to focus resources for monitoring and health risk assessment on gasoline-related VOCs of greatest concern. Additionally, a number of VOCs were not evaluated in the screening assessment because they did not have available toxicity values. Applying read-across approaches using structure-activity analyses and non-conventional toxicology data sets could help fill this data gap.

We were also limited in our ability to assess exposure potential and health concerns for most gasoline-related atmospheric transformation products of VOCs. Our analysis estimated that some of these chemicals, such as PAN and PAN analogs and a number of carbonyls, are among the most highly emitted gasoline-related chemicals when secondary formation is taken into account [46]. Short-term pilot studies could measure some of the transformation products of greatest potential concern to examine current ambient air levels and help determine if long-term monitoring would be warranted.

Based on measured ambient air data and supplementary modeling, we found that naphthalene was the gasoline-related PAH with the highest concentrations in the South Coast Air Basin over the time period assessed. We also found that gasoline-related emissions of naphthalene declined by 61% between 1996 and 2012, to 0.2 tons per day in the South Coast Air Basin in 2012. However, the gasoline-attributable cancer risk for naphthalene in the South Coast Air Basin in 2014 was still 4.8×10^-6^. This is about an order of magnitude higher than was estimated for other gasoline-related PAHs. Additional ambient air monitoring of naphthalene in areas impacted by gasoline-related pollution would be valuable to better characterize potential exposures and health risks.

OEHHA described a preliminary assessment of gasoline-related exposures to the criteria air pollutants nitrogen dioxide and PM_2.5_, but there was insufficient information to estimate gasoline-related health risks with confidence [46]. A study in California covering the period 1994 to 2014 found that decreases in nitrogen dioxide and PM_2.5_ were associated with improved lung function and decreases in the incidence of asthma in children [6, 73]. This association could be further investigated through research that better characterizes the contributions of gasoline-related sources to ambient particulate matter, particularly the ultrafine and secondary components, and nitrogen dioxide exposures [74]. The analysis could be extended to an evaluation of other potential health impacts, such as mortality, from gasoline-related exposures to PM_2.5_ and nitrogen dioxide (see, for example [75, 76]).

The screening methods we used to estimate average exposure and health risk for broad regions of the state do not address the potentially higher levels of gasoline-related air pollution in neighborhoods near highways and other heavily trafficked roads. We are conducting further work to evaluate gasoline-related air pollution in communities identified by CalEnviroScreen [77] as disproportionately burdened by environmental, socioeconomic and health stressors. One way we are approaching this is by measuring stable metabolites of VOCs and PAHs in urine samples from people living in communities heavily impacted by vehicle traffic. We are also measuring biomarkers of inflammation and oxidative stress, which provide another indication of air pollution exposures in these communities. This biomonitoring research is aiding in the understanding of hyperlocal exposures to air pollutants and advancing the goals of CARB’s Community Air Protection Program, established under Assembly Bill 617 (Garcia, Chapter 136, Statutes of 2017).

As vehicle emissions have declined, other sources, such as recreational boats, lawn mowers and all-terrain vehicles, have become more important contributors to gasoline-related emissions. Additional air quality improvements are being sought in California through further tightening of standards for small off-road engines, including those used in lawn and garden equipment [78]. California’s current dependence on gasoline-fueled transportation means the state will also need to maintain its efforts to address public health impacts from vehicle-related pollution until the transition to zero-emission vehicles planned by 2035 is implemented.

This historical analysis demonstrated the success of our state’s long-time efforts to improve air quality by showing significant decreases in gasoline-related pollutants of concern. California’s ongoing commitment to scientific research, forward-thinking regulations, and community-based approaches will be essential for continuing to reduce air pollution impacts.

### Supplementary information

Supplementary Material

## Data Availability

California air monitoring data for TACs and NMOCs can be obtained from the CARB by emailing AQMIS@arb.ca.gov. The NATTS dataset can be downloaded from the US EPA’s Ambient Monitoring Archive for HAPs. The Emission Inventory extracts used for this study were prepared by CARB staff. Inquiries about Emission Inventory data can be directed to eibweb@arb.ca.gov.
