# Supplementary material for: Analysis of gasoline-related pollutant exposures and risks in California between 1996 and 2014
Source: J Expo Sci Environ Epidemiol. 2023 Dec 8;34(3):518–28. doi: 10.1038/s41370-023-00615-0 (PMC11222143; doi:10.1038/s41370-023-00615-0)
Supplement: Supplementary file 1 — Supplementary Material [file 41370_2023_615_MOESM1_ESM.pdf]

Supplementary Material

Analysis of Gasoline-Related Pollutant Exposures and Risks in California Between 1996 and 2014

Daniel Sultana† and Sara Hoover†

Authors' affiliations
†Office of Environmental Health Hazard Assessment, California Environmental Protection Agency, Oakland, California, United States

***Corresponding author:**

Daniel Sultana
daniel.sultana@oehha.ca.gov

Table of Contents

Example Cancer Risk Calculation and Cancer Potencies for Assessed VOCs 3

Non-Cancer Health Reference Values 5

List of Top 100 Gasoline-Related Chemicals Based on 1996 Primary Emissions.........................6

Example Cancer Risk Calculation and Cancer Potencies for Assessed VOCs

The method for calculating the gasoline-attributable cancer risk for volatile organic compounds (VOCs) is demonstrated using acetaldehyde as an example. The gasoline-attributable population-weighted ambient air concentration of acetaldehyde was 1.98×10^{-3} mg/m^3 in the South Coast Air Basin in 1996.

The average daily dose (ADD) for each age range was calculated using the formula:

$$ADD_{age\ range} = (\text{Gasoline-attributable air concentration}) \times (\text{Breathing rate}_{age\ range})$$

Table S1 lists the factors for this example calculation and the resulting ADDs. The 95th percentile breathing rates adjusted for body weight within each age range were obtained from OEHHA (2015).

Table S1. Example calculation of acetaldehyde ADDs

**Age range**	**Gasoline-attributable air concentration (mg/m^3)**	**Breathing rate$_{age\ range}$ (m^3/kg-day)**	**ADD$_{age\ range}$ (mg/kg-day)**
3rd trimester	0.00198	0.361	7.15×10^{-4}
0<2 years	0.00198	1.090	2.16×10^{-3}
2<16 years	0.00198	0.745	1.48×10^{-3}
16<70 years	0.00198	0.290	5.74×10^{-4}

Cancer risk was calculated using the following formula:

$$\text{Cancer risk} = \sum_{age\ range} [(ADD_{age\ range} \times CPF \times ASF_{age\ range} \times [\text{fraction of lifetime})]$$

where $ADD_{age\ range}$ is the average daily dose for the age range, CPF is the cancer potency factor, $ASF_{age\ range}$ is the age sensitivity factor, and fraction of lifetime (unitless) is calculated as duration of age range divided by 70. Table S2 lists the factors for this example calculation and the resulting acetaldehyde cancer risk. ASFs were obtained from OEHHA (2015).

Table S2. Example calculation of acetaldehyde cancer risk

Age range	ADDage range (mg/kg-day)	CPF (mg/kg-day)$^{-1}$	ASF (unitless)	Duration (years)	Cancer risk (unitless)
3rd Trimester	7.15 x10^{-4}	0.010	10	0.3	3.1 x10^{-7}
0<2 years	2.16 x10^{-3}	0.010	10	2	6.2 x10^{-6}
2<16 years	1.48 x10^{-3}	0.010	3	14	8.9 x10^{-6}
16<70 years	5.74 x10^{-4}	0.010	1	54	4.4 x10^{-6}
				Sum	2.0 x10^{-5}

Table S3 lists cancer potencies for VOCs assessed in this paper (OEHHA 2015; 2010).

Table S3. Cancer potencies for assessed VOCs

Chemical	CPF (mg/kg-day)$^{-1}$
Acetaldehyde	1.0x10^{-2}
Benzene	1.0 x10^{-1}
1,3-Butadiene	6.0 x10^{-1}
Ethylbenzene	8.7 x10^{-3}
Formaldehyde	2.1 x10^{-2}
Methyl *t*-butyl ether (MTBE)	1.8 x10^{-3}
Naphthalene	1.2 x10^{-1}
Styrene	2.6 x10^{-2}

References

OEHHA (Office of Environmental Health Hazard Assessment). 2010. Public Health Goals for Chemicals in Drinking Water: Styrene. Available from: https://oehha.ca.gov/media/downloads/water/chemicals/phg/122810styrene_0.pdf

OEHHA. 2015. Air Toxics Hot Spots Program Guidance Manual for the Preparation of Risk Assessments. Available from: https://oehha.ca.gov/air/crnr/notice-adoption-air-toxics-hot-spots-program-guidance-manual-preparation-health-risk-0

Non-Cancer Health Reference Values

We primarily used chronic Reference Exposure Levels (cRELs) developed by the Office of Environmental Health Hazard Assessment (OEHHA) for the non-cancer health assessment. These are listed in Table S4 and available at https://oehha.ca.gov/air/general-info/oehha-acute-8-hour-and-chronic-reference-exposure-level-rel-summary.

Table S4. List of cRELs

**Chemical**	**cREL (µg/m^3)**	**Hazard Index Target Organs**
Acetaldehyde	140	Respiratory system
Acrolein	0.35	Respiratory system
Benzene	3	Hematologic system
1,3-Butadiene	2	Reproductive system
Ethylbenzene	2,000	Alimentary system (liver); kidney; endocrine system; development
Formaldehyde	9	Respiratory system
n-Hexane	7,000	Nervous system
Methyl *t*-butyl ether (MTBE)	8,000	Kidney; eyes; alimentary system (liver)
Naphthalene	9	Respiratory system
Propylene	3,000	Respiratory system
Styrene	900	Nervous system
Toluene	420	Eyes[1]
m-, *o*- *and p*-Xylene	700	Nervous & respiratory systems; eyes

[1]The underlying effect is acquired color vision impairment (dyschromatopsia), which reflects neural alterations in the peripheral nervous system. The toluene cREL is considered protective for neurotoxicity.

We relied on the US Environmental Protection Agency (US EPA, 1997; 2016) reference concentrations (RfCs) of 40 µg/m^3 for cumene (toxicity to endocrine and urinary systems) and 60 µg/m^3 for trimethylbenzenes (increased pain sensitivity). OEHHA (2023) developed a draft cREL of 4 µg/m^3 for trimethylbenzenes with the nervous system as the target organ. The gasoline-attributable hazard quotients for trimethylbenzenes were below 1 using either the RfC or the new draft cREL, as were the nervous system hazard indices.

References:

OEHHA. 2023. Trimethylbenzenes Reference Exposure Levels Technical Support Document for the Derivation of Noncancer Reference Exposure Levels Appendix D1 January 2023 Public Review Draft. Available at: https://oehha.ca.gov/media/downloads/crnr/tmbreldraft012723.pdf

US EPA (1997). Toxicological Review of Cumene. Available at: https://cfpub.epa.gov/ncea/iris/iris_documents/documents/toxreviews/0306tr.pdf

US EPA (2016). Toxicological Review of Trimethylbenzenes (Final Report). Available at: https://cfpub.epa.gov/ncea/iris/iris_documents/documents/toxreviews/1037tr.pdf

Table S5. List of Top 100 Gasoline-Related Chemicals Based on 1996 Primary Emissions. Includes primary emissions from 1996 and 2012, with the percent change between those two years.

**Chemical Name**	**1996 Primary emissions rank**	**1996 Primary emissions (tons per day)**	**2012 Primary emissions (tons per day)**	**Percent change in gasoline-related emissions between 1996 and 2012**
Isopentane	1	282.3	109.7	-61%
Methane	2	142.9	37.0	-74%
Methyl *t* -butyl ether (MTBE)	3	142.7	0.0	-100%
Toluene	4	130.6	38.3	-71%
Ethylene	5	122.7	31.3	-75%
n -Butane	6	117.5	20.2	-83%
n -Pentane	7	89.0	27.6	-69%
2-Methylpentane	8	87.1	29.9	-66%
m- Xylene	9	82.2	22.3	-73%
Propylene	10	65.1	16.2	-75%
Isobutylene	11	57.2	7.7	-87%
Methylcyclopentane	12	56.9	17.2	-70%
Benzene	13	53.3	13.9	-74%
Acetylene	14	52.7	12.1	-77%
3-Methylpentane	15	50.6	17.3	-66%
2,2,4-Trimethylpentane	16	39.4	25.6	-35%
n- Hexane	17	35.6	10.8	-70%
Formaldehyde	18	35.5	8.8	-75%
Isobutane	19	35.4	3.6	-90%
2,3-Dimethylpentane	20	32.9	15.4	-53%
o- Xylene	21	28.5	8.8	-69%
2,3-Dimethylbutane	22	26.9	9.6	-64%
Ethylbenzene	23	25.4	8.4	-67%
Ethane	24	23.6	5.8	-76%
1,2,4-Trimethylbenzene	25	22.0	7.2	-68%
3-Methylhexane	26	19.2	12.9	-33%
1-Methyl-3-ethylbenzene	27	18.0	5.8	-68%
Propane	28	16.2	1.3	-92%
2,2-Dimethylbutane	29	15.4	6.0	-61%
2-Methyl-2-butene	30	15.2	4.9	-68%
Cyclohexane	31	14.6	4.3	-70%
Methylcyclohexane	32	14.0	5.4	-61%
2,3,4-Trimethylpentane	33	13.8	7.2	-48%
Methyl alcohol	34	13.4	1.1	-92%
2,4-Dimethylpentane	35	13.1	8.2	-38%
n- Heptane	36	12.7	5.7	-55%
3-Methylheptane	37	11.4	4.8	-57%
1,3-Butadiene	38	11.4	2.9	-75%
Cyclopentane	39	10.8	5.2	-51%
1-Butene	40	10.4	2.6	-75%
trans -2-Pentene	41	9.8	3.2	-67%

Chemical Name	1996 Primary emissions rank	1996 Primary emissions (tons per day)	2012 Primary emissions (tons per day)	Percent change in gasoline-related emissions between 1996 and 2012
trans -2-Butene	42	9.3	2.5	-73%
2-Methyl-1-butene	43	9.2	2.1	-77%
1,3,5-Trimethylbenzene	44	8.7	2.8	-68%
Acetaldehyde	45	7.9	2.8	-65%
1-Methyl-4-ethylbenzene	46	7.8	2.5	-68%
cis -2-Butene	47	7.5	1.8	-76%
n -Octane	48	7.5	2.6	-65%
2-Methylheptane	49	7.4	3.7	-50%
2,5-Dimethylhexane	50	7.3	3.4	-53%
3-Methyloctane	51	6.8	2.4	-65%
2,4-Dimethylhexane	52	6.6	3.9	-41%
2,2,5-Trimethylhexane	53	6.5	3.7	-43%
3-Ethylpentane	54	6.4	1.9	-70%
1-Methyl-2-ethylbenzene	55	6.2	1.9	-69%
trans -1,3-Dimethylcyclopentane	56	6.2	2.5	-60%
Tolualdehyde	57	6.1	1.7	-72%
2-Methylhexane	58	6.1	9.6	57%
Benzaldehyde	59	6.0	1.7	-71%
2,3-Dimethylhexane	60	5.7	2.9	-49%
cis -1,3-Dimethylcyclopentane	61	5.6	2.4	-56%
1-Propyne	62	5.5	1.4	-74%
Acetone	63	5.2	1.5	-72%
n -Propylbenzene	64	5.1	1.6	-69%
cis -2-Pentene	65	4.9	1.5	-69%
3-Methyl-1-butene	66	4.7	1.7	-63%
1-Pentene	67	4.6	1.3	-71%
4-Methyloctane	68	4.4	1.4	-68%
1,2,3-Trimethylbenzene	69	4.1	1.3	-68%
Cyclopentene	70	4.1	1.1	-73%
1,2-Propadiene	71	4.0	1.1	-73%
1-Methyl-3-*n* -propylbenzene	72	3.9	1.2	-68%
4-Methylheptane	73	3.8	1.7	-54%
n -Nonane	74	3.6	1.1	-70%
2,6-Dimethylheptane	75	3.4	0.8	-75%
Ethylcyclopentane	76	3.2	1.3	-59%
trans -2-Hexene	77	3.1	0.9	-72%
3,5-Dimethylheptane	78	2.9	0.7	-75%
Isoprene	79	2.8	0.7	-77%
1,2,4-Trimethylcyclopentene	80	2.8	0.6	-79%
2-Methyl-2-pentene	81	2.7	0.9	-65%
1,3-Dimethyl-5-ethylbenzene	82	2.7	0.9	-67%
1,2-Dimethyl-4-ethylbenzene	83	2.5	0.9	-64%
2-Methylnonane	84	2.5	0.7	-74%

Chemical Name	1996 Primary emissions rank	1996 Primary emissions (tons per day)	2012 Primary emissions (tons per day)	Percent change in gasoline-related emissions between 1996 and 2012
Styrene	85	2.5	0.6	-75%
Acrolein	86	2.4	0.6	-75%
n- Decane	87	2.2	0.5	-76%
2-Methyl-2-propenal	88	2.2	0.6	-73%
Ethanol	89	2.2	39.0	1697%
Indan	90	2.2	0.7	-70%
trans -1-Methyl-3-ethylcyclopentane	91	2.1	0.5	-75%
cis -1,3-Dimethylcyclohexane	92	1.9	0.5	-74%
3-Methyl-1-pentene	93	1.9	0.4	-80%
2,2,5-Triethylheptane	94	1.8	0.4	-77%
3-Methylcyclopentene	95	1.8	0.4	-78%
1,4-Diethylbenzene (*para*)	96	1.7	0.4	-75%
Vinylacetylene	97	1.7	0.4	-75%
1,3-Dimethyl-4-ethylbenzene	98	1.6	0.6	-64%
2-Methyl-1-pentene	99	1.6	0.4	-73%
1,4-Dimethyl-2-ethylbenzene	100	1.6	0.6	-65%
